# Socioeconomic and Demographic Factors Effect in Association with Driver’s Medical Services after Crashes

**DOI:** 10.3390/ijerph19159087

**Published:** 2022-07-26

**Authors:** Shraddha Sagar, Nikiforos Stamatiadis, Rachel Codden, Marco Benedetti, Larry Cook, Motao Zhu

**Affiliations:** 1 University of Florida Transportation Institute, University of Florida, Gainesville, FL 32611, USA; 2 Department of Civil Engineering, University of Kentucky, Lexington, KY 40506, USA; nick.stamatiadis@uky.edu; 3 School of Medicine, University of Utah, Salt Lake City, UT 84112, USA; rachel.codden@hsc.utah.edu; 4 Center for Injury Research & Policy, Abigail Wexner Research Institute at Nationwide Children’s Hospital, Columbus, OH 43215, USA; marco.benedetti@nationwidechildrens.org (M.B.); motao.zhu@nationwidechildrens.org (M.Z.); 5 Department of Pediatrics, University of Utah, Salt Lake City, UT 84112, USA; larry.cook@hsc.utah.edu

**Keywords:** general use model, highway safety, socioeconomic factors, logistic regression

## Abstract

Motor vehicle crashes are the third leading cause of preventable-injury deaths in the United States. Previous research has found links between the socioeconomic characteristics of driver residence zip codes and crash frequencies. The objective of the study is to extend earlier work by investigating whether the socioeconomic characteristics of a driver’s residence zip code influence their likelihood of resulting in post-crash medical services. Data were drawn from General Use Model (GUM) data for police crash reports linked to hospital records in Kentucky, Utah, and Ohio. Zip-code-level socioeconomic data from the American Community Survey were also incorporated into analyses. Logistic regression models were developed for each state and showed that the socioeconomic variables such as educational attainment, median housing value, gender, and age have *p*-values < 0.001 when tested against the odds of seeking post-crash medical services. Models for Kentucky and Utah also include the employment-to-population ratio. The results show that in addition to age and gender, educational attainment, median housing value and rurality percentage at the zip code level are associated with the likelihood of a driver seeking follow-up medical services after a crash. It is concluded that drivers from areas with lower household income and lower educational attainment are more likely to seek post-crash medical services, primarily in emergency departments. Female drivers are also more likely to seek post-crash medical services.

## 1. Introduction

Motor vehicle crashes in the US are the third leading cause of preventable injury-related deaths. In Kentucky, they rank as the second leading cause of injury-related deaths, and in Utah and Ohio, third. Deaths per 100 million vehicle miles traveled (VMT) in Kentucky have exceeded the national average since 1986 [1,2]. Previous research has demonstrated that socioeconomic and demographic characteristics of the zip codes in which drivers reside influence their involvement in crashes [3,4]. Past research investigated the effect of socioeconomic and demographic factors associated with the residence location of the drivers involved in a crash, using them as a surrogate descriptor [3,4,5] and using the socioeconomic data from the drivers’ residence zip codes [6]. Income, poverty, employment, education, rurality, and driver age all appear to influence crash propensity [4,7,8,9,10,11]. Previous research showed a well-defined relationship between educational attainment and crashes [3,7,12]. Race is a potential contributing factor as well [7]; however, research on the link between race and crashes is sparse. Another robust predictor of crash propensity is driver marital status [11]. Drivers who live in lower household income areas are more likely to be involved in a crash and be found as the at-fault party [4]. Age is another influence on crash propensity. Chen et al. [13] and Factor et al. [11] showed that young or new drivers are involved in more crashes and have higher fatality rates than other age groups.

Several research efforts have identified variables that can potentially impact crash occurrence and severity. While police-issued crash reports include data on severity, they are completed at the scene before full medical examinations are performed. This has motivated efforts to link crash records with hospital data to obtain more accurate and complete information on injuries and crash severity. For example, individuals involved in what police characterize as a possible injury crash (i.e., complaints of pain without visible injury) could result in post-crash medical services after experiencing discomfort. Had police known this information, they may have upgraded the crash severity. Integrating linked crash and hospital records into safety analyses can provide a more comprehensive view of how resources need to be allocated to prevent fatalities, reduce injury severity, and lower healthcare costs.

Such efforts began in 1996 when the National Highway Traffic Safety Administration (NHTSA) established the Crash Outcome Data Evaluation System (CODES) program [14]. The CODES Data Network developed a standardization model that maps state-specific crash files onto a standardized format called the General Use Model (GUM). Probabilistic linkages were used to combine information from motor vehicle crash reports and several types of medical data to develop GUM datasets. Integrating linked crash and hospital records into safety analyses can provide a more comprehensive view of how resources need to be allocated to prevent fatalities, reduce injury severity, and lower healthcare costs. Several previous research efforts utilized CODES data for studies such as injury assessments for bicycle and pedestrian crashes [15], seat belt studies [16] and helmet usage [17]. These research efforts have demonstrated the benefits of using linked data to estimate crash severity and improve driver safety.

While socioeconomic status is clearly associated with health condition and healthcare utilization, there has been limited research along this line. The current study investigates the socioeconomic factors associated with the driver’s residence zip code that could influence the likelihood of the driver seeking post-crash medical services. Using data from Kentucky, Utah, and Ohio, this study investigates whether the socioeconomic conditions of the zip code in which a driver resides influence their likelihood of being involved in a crash that results in seeking post-crash medical services. Data were drawn from the GUM dataset that has linked police crash reports and hospital records. Measuring correlations between driver zip code socioeconomic characteristics and the need for seeking post-crash medical attention will improve our understanding of the factors most likely to identify those seeking post-crash medical services.

A person’s socioeconomic status significantly impacts their healthcare service utilization [17]. Although healthcare delivery in the US has been transformed in recent years, the availability of newer and improved healthcare services does not guarantee equal availability of services to all people. A person’s socioeconomic status significantly impacts their healthcare service utilization [18,19]. For example, Medicaid recipients are more likely to use emergency departments (EDs) than people who have other coverage as they have less access to ambulatory care [19]. Some research has found that poorer individuals are more likely to have a sedentary or unhealthy lifestyle and therefore are more likely to suffer poor health outcomes or endure disabling conditions than wealthier people who have greater access to resources [18,19]. This research investigates the association between a person’s socioeconomic status and healthcare utilization post involvement in a crash. Practitioners can utilize the output of this research to propose potential improvements in health insurance policies.

## 2. Materials and Methods

### 2.1. Dataset Development

This study leveraged the GUM data from Kentucky, Ohio and Utah that linked motor vehicle crash, ED, and hospital discharge records. The analysis encompasses single and multi-vehicle crashes during the study period. Study periods varied by state—Kentucky, 2008–2014; Ohio 2009–2016; and Utah 2008–2018.

Information on driver residence zip codes was extracted from the state police databases and merged with GUM datasets. Data were cleaned to remove invalid entries. The final dataset consisted of 928,692 driver records for Kentucky, 3,078,229 for Ohio and 1,069,777 for Utah and included drivers between 15 and 90 years old. For this analysis, drivers were divided into six age groups—<20, 20–24, 25–39, 40–64, 65–74 and >74 years. The drivers were grouped in these groups to conform to the socioeconomic data. A representative control dataset was created by drawing from the unlinked records a random sample equal to the number of hospital-linked records. The sampling procedure ensured an equal distribution of crash severities between the linked and unlinked data. Table 1 summarizes the data from each state and indicates the number of cases for linked and unlinked driver records.

### 2.2. Socioeconomic Data

As discussed above, several studies show that socioeconomic and demographic factors associated with the residence location of the drivers can be used as a surrogate measure to describe crash occurrence. Income, poverty, employment, education, rurality, and driver age are some of the factors that influence crash propensity. As an attempt to investigate the association of these factors on healthcare service utilization post involvement in a crash, several socioeconomic and demographic factors were selected based on the recommendations from prior research findings. The list of variables selected and examined in this study is presented in Table A1 in Appendix A.

Several zip-code-level socioeconomic data for all three states were extracted from the American Community Survey [20] data. The data collected were variables associated with Race, Housing, Marital Status, Education, Income and Others (such as employment by population ratio, percent rural etc.). The data were combined with each state’s GUM dataset. Additional socioeconomic variables were derived from Census data. The proportion of the population eligible to drive was calculated and incorporated as an explanatory variable. Driver population density for each zip code was calculated using measured zip code areas. Data on driver age and gender were extracted from crash data.

### 2.3. Statistical Analysis

The response variable of the study is whether a driver sought medical services post involvement in a crash, while the predictor variables are the socioeconomic characteristics of the driver’s residence zip code. Logistic regression was selected because the response variable is whether a driver’s crash record is linked to a hospital database and is categorical.

In logistic regression, the response variable’s expected values are modeled based on the probability—or odds—of it taking a particular value based on combinations of predictor values. The natural logarithm of the odds (i.e., log-odds or logit function) is defined in Equation (1). Here, p is the probability of a driver seeking post-crash medical services

(1) $$\mathrm{logit}\;(p)=\ln\;\left(\frac{p}{1-p}\right)=f\left(X\right)$$

The probability takes its final form as such:

(2) $$p=\frac{1}{1+e^{-f\left(X\right)}}$$

where *f*(*X*) = a + b_1_ *X*_1_ + b_2_ *X*_2_ + … + b_n_ *X*_n_ is the regression model, *X*i is the ith explanatory variable, a is the intercept and bi is the ith coefficient estimated using the maximum likelihood method.

### 2.4. Model Development Process

Correlation and recursive partitioning analysis served as the starting point for logistic regression by winnowing potential explanatory variables. Correlation was used to explore relationships between the response variable and socioeconomic variables. Recursive partitioning analysis was also used to understand the association between explanatory and response variables. The method generates an importance score that captures the relative importance of explanatory variables in predicting the response variable. When building logistic regression models, the outputs from the two analyses were utilized.

Several potential interactions between the socioeconomic variables might influence crash occurrence. Potential interactions were identified using R’s Shiny package, which employs the Feasible Solution Algorithm (FSA) to detect statistical interactions in large datasets [21]. This approach enables the formulation of new models or improvements to existing models. FSA allows higher-order interactions; however, for this study, two-way interactions are used.

## 3. Results

### 3.1. Correlation Test

**Kentucky** All racial categories, except for the percent of zip code identifying as white, were significantly but weakly correlated with the response variable. Median housing value, marital status, and educational attainment were also significantly correlated. Drivers from zip codes with lower educational attainment were more likely to be involved in a crash and seek post-crash medical services; percentage of residents in a zip code with less than a high school degree was identified as a potential candidate for describing education-related effects. All income levels were significantly related to the response variable, as were all variables in the Other category, although employment by population showed a stronger association. Driver age and gender have a well-established relationship with crash occurrence, qualifying them as potential predictor variables.

**Ohio** All explanatory variables were weakly correlated with the response variable. Education variables returned the highest correlations with a negative correlation with the response variable. Three candidate explanatory variables were not statistically significantly correlated with seeking post-crash medical services—driver population, renter occupied housing units and area of zip code.

**Utah** Data from Utah yielded results similar to Kentucky. Weak correlations were observed between the candidate and the response variables. The variables displaying the highest absolute correlations were percent bachelor’s degree or higher, median housing value, and mean individual income. Most correlations were statistically significant. Only three lacked a statistically significant relationship with the response variable—percent white, percent other races, and renter-occupied housing units. All variables in the marital, education, income and Other categories were statistically significantly correlated with the response variable.

### 3.2. Recursive Partitioning Analysis

Recursive partitioning analysis identified several factors, including education, housing value, rurality, employment, and income, as possible explanatory variables. Table A2 in the Appendix A lists the recursive partitioning analysis for the variables deemed most important for each state.

In all states, gender ranked as the highest or second highest predictive variable. Age was also of high importance, along with some socioeconomic variables. Among the zip-code-level economic predictors, median individual income ranked highly in all states, while median housing value was highly important in Kentucky and Ohio. Two predictors related to educational attainment—the percentage of residents with at least a bachelor’s degree and the percentage with at least some high school education—had high importance. In all states, the percentage of rural areas in each zip code was of high importance. In short, the recursive partitioning analysis suggested the potential for developing state-specific models using common variables, thus facilitating comparisons.

### 3.3. Final Model

For each state, several models were tested and evaluated (using goodness-of-fit measures such as AIC, BIC, AUC and percent correctly predicted) to select a final model. A training and validation approach was used for the model development to estimate accuracy in predicting the response variable—20% of observations were placed in the training dataset and 80% in the validation dataset. Along with the main effects, possible interactions were explored to improve each model’s mathematical stability. Table 2 lists the parameters of each state’s final model. All variables were significant (*p* < 0.001) except those noted in bold. Models were tested for potential interactions between variables. No statistically significant interactions were identified. Training and validation analysis found that the models predict approximately 57% of the data correctly in each state.

All state models include zip-code-level socioeconomic variables such as percent with bachelor’s degree (BS), median housing value (HVL), and percentage rural (RUR). Gender and age are common variables as well. Models for Kentucky and Utah also incorporate employment-to-population ratio (EMP). Socioeconomic predictors (e.g., BS, HVL, EMP) are negatively related to the response variable, while RUR exhibits a positive association. The impact of these socioeconomic factors on crashes is supported by several re-search studies but is not limited to Hasselberg et al., 2005 [3], Sagar et al., 2021 [4] Noland et al., 2004 [5], and Adanu et al., 2017 [7].

Table A3 in Appendix A shows the summary statistics of the continuous predictor variables in the models for all three states.

**Figure 1:**
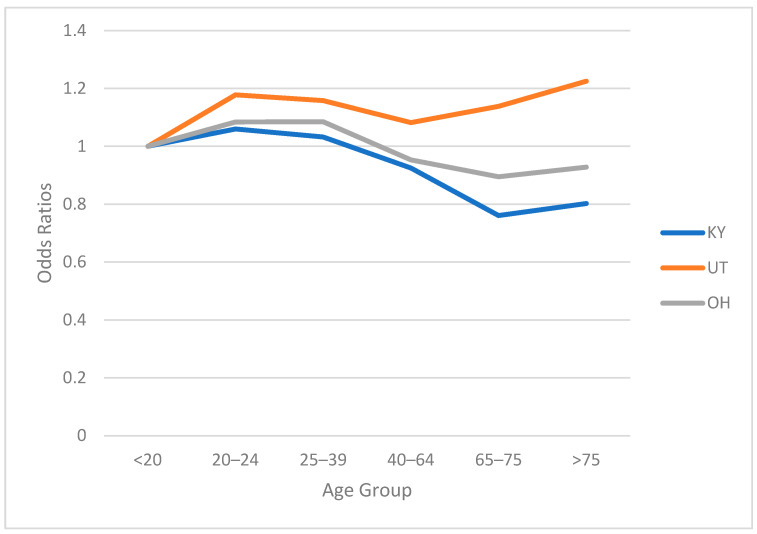
Odds ratio for age groups.

The odds ratio for gender indicates that female drivers are more likely to be involved in a crash seeking post-crash medical services. Figure 1 shows the odds ratio for different age groups in each of the three states. Odds ratios suggest that drivers in the age category of 20–24 years are more likely to be involved in a crash resulting in medical services. This trend decreases with age before increasing for older groups. The inflection point differs by state. In Kentucky and Ohio, the reversal occurs for drivers 65 and older, while in Utah, the threshold is 40 years of age. 

## 4. Discussion

This research is one of the first studies that attempts to correlate the socioeconomic factors of a driver involved in a crash seeking post-crash medical services. This study confirms that drivers living in zip codes with lower housing values are more likely to seek post-crash medical services.

One explanation is that residents of lower-income zip codes are more likely to have more pre-existing conditions [22] and therefore suffer greater impacts when involved in a motor vehicle crash. Ukert et al. [23] discuss the disparities in healthcare use among low- and high-income communities. They concluded that HDHPs (High Deductible Health Plans) discourage routine checkups among low-salary employees. It is scientifically established that the disparities in healthcare utilization differ between communities of different socioeconomic statuses [24,25,26]. The impact of these socioeconomic factors on crashes is supported by several research studies but is not limited to Hasselberg et al., 2005 [3], Sagar et al., 2021 [4] Noland et al., 2004 [5], and Adanu et al., 2017 [7].

Another reason is that persons with lower socioeconomic status are more likely to use EDs. Current healthcare delivery in the US does not guarantee equal availability of services to all people. A person’s socioeconomic status significantly impacts their healthcare service utilization [18,19]. Research has documented that Medicaid recipients are more likely to use EDs than people who have other coverage [19]. In the Kentucky and Utah GUM data, most of the linked records (85% for Kentucky, 91% for Utah) are from ED admissions, which substantiates the assertion that drivers from lower economic areas are over-represented in the database.

Overreliance on ED services is a serious issue in the US. Many patients who visit EDs do not require urgent services [27]. Ukert et al. [23] showed that lower-salary employees in HDHPs underutilize outpatient care and overutilize EDs. These individuals may lack or be unaware of other care options and depend on ED services for non-urgent problems. Bilings et al. [28] suggested that changing the way people use EDs will require improvements to the primary care system. Potential solutions to this dilemma include expanding the availability of affordable health insurance coverage, improving access to primary healthcare services, and making telemedicine consultations more accessible. Primary care clinics can be better rewarded for providing a lower-cost alternative to ED use and for preventing emergency situations from developing [28].

Discrepancies in age trends between Utah, Kentucky and Ohio could reflect differences in driver populations and characteristics. By area, Utah is the thirteenth largest state in the US; however, beyond the Interstate 15 corridor, the state is sparsely populated (ACS) and has a population density of 39.01 people per square mile. The state has a low percentage of older drivers (11.9%) and the youngest median age in the US. Conversely, Kentucky’s population density is 113.12 persons per square mile, and 16.9% of the population is classified as older, while Ohio’s population density is 286.13 persons per square mile, and 17.5% of the population is older. These differences between Utah and the other two states could explain the dissimilar age trends shown in Figure 1.

## 5. Conclusions

The mathematical model developed in the study attempted to correlate the socioeconomic factors of a driver involved in crash seeking post-crash medical services. The study concludes that (1) drivers residing in areas with lower socioeconomic status and (2) female drivers are more likely to seek post-crash medical services.

This study is one of the few studies that have investigated the relationship of the drivers’ socioeconomic factors with their seeking of post-crash medical services. However, the study carries several limitations. First is its dependence on GUM data, which primarily uses police-reported crash records. Upwards of 10 million crashes go unreported each year [29]. Furthermore, some drivers might seek medical attention regardless of injury severity. Crashes that do not appear in crash records are not traceable and thus excluded from analysis, which could potentially bias the findings. However, this is unavoidable. The linkage approach used to develop the GUMs data is probabilistic and imperfect. Furthermore, this study used socioeconomic data derived from the ACS at the zip code level and assumed that information observed at the zip code level also holds true at the individual level. When studies are conducted at the group level, this ecological fallacy is common and irresolvable. Crash types were not factored into the analysis presented here, and future research should take a closer look at the relationship between crash type and crash severity, as this may contribute to whether a driver seeks medical attention.

## Figures and Tables

**Table 1:** Distribution of age groups.

Age Groups	Kentucky		Utah		Ohio	
	Linked	Unlinked	Linked	Unlinked	Linked	Unlinked
<20	38,889	40,727	7831	8559	36,403	36,915
20–24	14,151	13,564	10,622	10,018 (48.4%)	46,721	53,460
25–39	16,845	15,557	22,530	21,856 (49.2%)	95,573	110,788
40–64	35,349	33,297	19,330	20,112 (51.0%)	127,640	125,907
65–74	5586	6997	3131	3063 (49.3%)	21,134	18,666
>75	3231	3909	1872	1708 (47.7%)	12,659	11,339
Total	114,051	114,051	65,316	65,316	357,146	357,146

**Table 2:** Final model; Kentucky, Utah and Ohio.

Variables	Kentucky			Utah			Ohio		
	B	S.E.	Odds	B	S.E.	Odds	B	S.E.	Odds
			Ratio			Ratio			Ratio
Age < 20	ref		1	ref		1	ref		1
Age 20–24	0.059	0.017	1.06	0.164	0.021	1.178	0.08	0.01	1.084
Age 25–39	**0.031**	0.014	1.032	0.147	0.019	1.158	0.082	0.008	1.085
Age 40–64	−0.078	0.014	0.925	0.079	0.019	1.082	−0.048	0.008	0.953
Age 65–75	−0.273	0.022	0.761	0.129	0.03	1.138	−0.111	0.015	0.895
Age > 75	−0.221	0.027	0.802	0.203	0.037	1.225	−0.074	0.019	0.928
Male	ref		1	ref			ref		1
Female	0.262	0.009	1.299	0.254	0.011	1.289	0.386	0.008	1.472
RUR ^1^	0.017	0	1.017	0.034	0.004	1.035	**1.80 × 10^−5^**	0	1
EMP ^1^	−0.103	0.001	0.99	−0.092	0.012	0.912	--		--
BS ^1^	−0.106	0.001	0.989	−0.09	0.014	0.914	−0.095	0.01	0.909
HVL ^2^	−0.016	0	0.984	−0.006	0.001	0.994	−0.015	0.002	0.985
Constant	0.732	0.036		0.669	0.083		0.257	0.013	

Legend: RUR: percentage rural; EMP: employment-to-population ratio; BS: percent with bachelor’s degree; HVL: median housing value. ^1^. Values are per 10%; ^2^. Values are per $10,000. All variables were significant (*p* < 0.001) except those noted in bold.

## Appendix A

**Table A1:** List of Socioeconomic and Demographic Variables.

Category	Variable	Category	Variable
Race	Percent white (WH)	Marital Status	Percent now married (MRD)
	Percent black (BL)		Percent widowed (WID)
	Percent American Indian (AI)		Percent divorced (DIV)
	Percent Asian (AS)		Percent separated (SEP)
	Percent other races (OR)		Percent never married (NMD)
Housing	Household units (HH)	Education	Percent less than high school graduate (LHS)
	Household ownership total (HHO)		Percent high school graduate (HS)
	Owner occupied housing units (OHU)		Percent some college/associate degree (COL)
	Renter occupied housing units (RHU)		Percent bachelor’s degree or higher (BS)
	Median housing value (HVL)		Percent graduate or professional degree (GD)
Other	Employment population ratio (EMP)	Income	Median individual income (MDIINC)
	Percentage rural (RUR)		Household median income (MDHINC)
	Unemployment rate (UEMP)		Household mean income (MHINC)
	Percent below poverty level (POV)		Mean individual income (MIINC)
	Total population (POP)		

**Table A2:** List of ACS Variables with High Importance.

Variables	Kentucky		Utah		Ohio	
	Relative	Importance	Relative	Importance	Relative	Importance
Percent bachelor’s degree or higher (BS)	1.000	33.969	0.543	9.292	0.667	22.4812
Gender	0.599	20.353	0.918	15.699	1.000	33.693
Median housing value (HVL)	0.558	18.944			0.356	12.005
Percent graduate or professional degree (GD)	0.532	18.069			0.134	4.527
Age Groups (AGE)	0.417	14.155	0.334	5.713	0.495	16.689
Percentage rural (RUR)	0.396	13.465	1.000	17.108	0.216	7.292
Employment population ratio (EMP)	0.335	11.364	0.267	4.497		
Percent white (WH)	0.230	7.817	0.083	1.412		
Percent high school graduate (HS)	0.206	7.005	0.178	3.046	0.186	6.275
Percent less than high school graduate (LHS)	0.194	6.587	0.553	9.454		
Percent some college/associate degree (COL)	0.193	6.553	0.188	3.209	0.264	8.883
Percent black (BL)			0.247	4.229	0.188	6.321

**Table A3:** Summary Statistics of Predictor Variables; Kentucky, Utah and Ohio.

State	Variables	Linked					Unlinked				
		N	Minimum	Maximum	Mean	Std. Deviation	N	Minimum	Maximum	Mean	Std. Deviation
Kentucky	RUR ^1^	114,051	0	10	4.45	3.80	114,051	0	10	3.73	3.66
	EMP ^1^	114,046	0	10	5.37	1.01	114,043	0	10	5.59	0.95
	BS ^1^	114,046	0	5.13	1.13	0.68	114,043	0	5.13	1.31	0.76
	HVL ^2^	113,363	2.14	38.53	12.22	4.90	113,484	2.14	38.53	13.44	5.42
Utah	RUR ^1^	65,316	0.87	10	1.03	1.71	65,316	0.87	10	0.87	1.59
	EMP ^1^	64,669	11.8	8.48	6.54	0.58	64,577	0	10.00	6.59	0.53
	BS ^1^	64,669	0	5.353	1.84	0.67	64,577	0	6.41	1.92	0.69
	HVL ^2^	113,363	2.91	101.5	25.59	8.48	113,484	2.91	101.5	26.60	9.09
Ohio	RUR ^1^	116,098	0	10	7.19	3.48	131,772	0	10	7.21	3.45
	EMP ^1^	NA	NA	NA	NA	NA	NA	NA	NA	NA	NA
	BS ^1^	116,073	0	5.33	1.47	0.79	131,772	0	5.33	1.58	0.88
	HVL ^2^	113,363	1.48	49.67	12.78	4.79	113,484	1.48	49.67	13.46	5.26

Notes: ^1^. Values are per 10 percent; ^2^. Values are per $10,000.
